# Posterior epidural migration of a lumbar disk: an entity not to ignore

**DOI:** 10.11604/pamj.2018.29.59.9492

**Published:** 2018-01-22

**Authors:** Samia Frioui, Faycel Khachnaoui

**Affiliations:** 1 Physical Medicine and Rehabilitation Department, Sahloul Hospital Sousse, Tunisia

**Keywords:** Lumbar disc herniation, disc migration, cauda equina syndrome, magnetic resonance imaging

## Image in medicine

Discal hernia with posterior epidural migration is exceptional. Its diagnosis is difficult and often takes the form of an intraspinal tumor lesion. It has a various clinical manifestations. The association of this entity to a cauda equina syndrome found as isolated cases which were reported in the literature. MRI with injection is the best diagnostic tool. We must always consider this diagnosis in front of a posterior epidural mass with a peripheral enhancement on the contrast-enhanced images. Emergency surgery is needed to prevent serious neurological deficits. We report the case of a 29-year old patient who presented an acute lumbo-sciatica associated with a deficit of both lower limbs and urinary incontinence after lifting a heavy load. Clinical examination revealed a motor deficit of the triceps surae and in the relievers of the foot rated 1 in bilateral sides, hypoesthesia at the anterolateral part of the two legs and the feet, weak or abolished tendon reflexes, anesthesia in the saddle, hypotonic anal sphincter and bladder distension. MRI of the lumbar spine showed a right lumbar discal hernia (L3-L4) excluded with right posterior epidural migration compressing the dural sheath. Surgical treatment was undertaken in emergency: a decompression and herniectomy were performed. A partial sensor-motor recovery was observed. After ten months, the patient still has had a distal deficit of both lower limbs, bladder sphincter disorders for which it is under self-intermittent catheterization.

**Figure 1:**
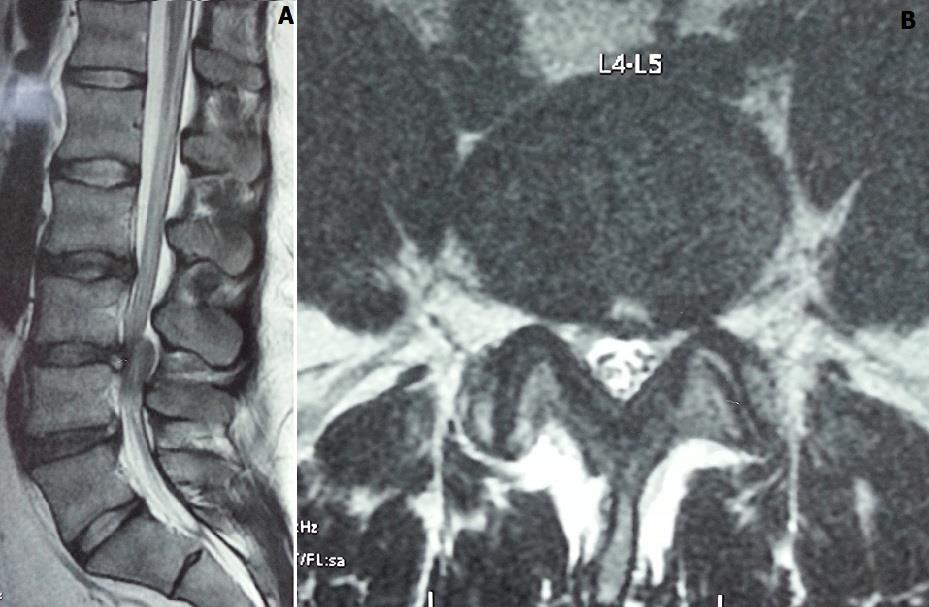
A) lumbar MRI sagittal section: mass oval shape about 2 cm height with same signal as the intervertebral disc intra ductal, extra dural seat, posterior epidural at the height of L3L4 intervertebral disk compressing the dural sheath on the same floor; B) lumbar MRI axial section: mass oval shape with same signal as the intervertebral disc intra ductal, extra dural seat, posterior epidural at the height of L3L4 intervertebral disk compressing the dural sheath on the same floor

